# mSphere of Influence: Frameshift—a Vision for Human Microbiome Research

**DOI:** 10.1128/mSphere.00944-20

**Published:** 2020-09-30

**Authors:** Ariangela J. Kozik

**Affiliations:** a Division of Pulmonary and Critical Care Medicine, Department of Internal Medicine, University of Michigan, Ann Arbor, Michigan, USA

**Keywords:** asthma, disparities, microbiome

## Abstract

Ariangela J. Kozik studies the respiratory microbiome as it relates to asthma. In this mSphere of Influence article, she reflects on how two papers, “Time’s up to adopt a biopsychosocial model to address racial and ethnic disparities in asthma outcomes” (E. C. Matsui, A. S. Adamson, and R. D. Peng, Allergy Clin Immunol 143:2024–2025, 2019, https://doi.org/10.1016/j.jaci.2019.03.015) and “Health disparities and the microbiome” (K. Findley, D. R. Williams, E. A. Grice, and V. L. Bonham, Trends Microbiol 24:847–850, 2016, https://doi.org/10.1016/j.tim.2016.08.001), shape her approach to human microbiome research.

## Abstract (precis)

Ariangela J. Kozik studies the respiratory microbiome as it relates to asthma. In this mSphere of Influence article, she reflects on how two papers, “Time’s up to adopt a biopsychosocial model to address racial and ethnic disparities in asthma outcomes” (E. C. Matsui, A. S. Adamson, and R. D. Peng, Allergy Clin Immunol 143:2024–2025, 2019, https://doi.org/10.1016/j.jaci.2019.03.015) and “Health disparities and the microbiome” (K.

## COMMENTARY

I have few vivid memories of my elementary school days. One is the smell. Some of the nation’s largest integrated steel mills, an oil refinery, several coal-fired power plants, lead smelters, toxics recyclers, chemical plants, and railyards stand within a 10-mile radius of the school. Every morning, even on sunny days, there was a thick blanket of haze that reeked of smoke, gasoline, and melting plastic. My other memory is how many of my northwestern Indiana school classmates went to the nurse’s office for their asthma medications each day.

Asthma is a chronic inflammatory disease of the respiratory system that affects adults and children. Minoritized populations, specifically Black and Hispanic persons, disproportionately experience adverse asthma outcomes. This increased burden is especially acute in pediatric asthma, with Black children two and four times as likely to be hospitalized or die from asthma than white children, respectively (1). Despite decades of asthma research, substantial progress toward understanding and mitigating these stark differences in outcomes has not been made. While the definitive cause of asthma is unknown, knowledge that the human microbiome is highly individualized, responsive to environmental exposures, and intricately connected to the immune system makes it a subject of research in this (2) and other chronic diseases (3, 4).

Studies have traditionally indicted the Black body for biological explanations of their disproportionate disease burden in the United States. Unsurprisingly, this framework has failed to meaningfully improve the lives and health of Black communities. This is the context within which two recent articles have influenced me in their call for a new approach to research. In their article “Time’s up to adopt a biopsychosocial model to address racial and ethnic disparities in asthma outcomes” (5), Matsui et al. discuss a study that matched Black and white subjects with asthma based on their community/family socioeconomic variables and environmental exposure variables, eliminating the racial disparity in asthma-related emergency department visits (6). In their discussion, they assert the need to develop research frameworks (especially research aimed at reducing racial disparities) that center the social determinants of health and biology. Without these considerations, the conventional biological models of racial disparities conflate race with biology, which fails to produce meaningful mechanistic insights or reduce disparities (7, 8). The authors affirm two important principles. (i) Race is a social construct, and it functions (in the United States) as a “conveniently measurable proxy” for other factors that influence health. It is critical to understand that the connections between race and these factors (health literacy, environment, social vulnerability, stress, etc.) did not occur by accident. They are the direct consequence of racism. The exploitative systems of racial classification were constructed to politically serve white supremacy. (ii) Conventional framing of biomedical studies that employ “race” as a potential causal factor actively ignores other factors that can shape the biology in a person or given population.

“Health disparities and the microbiome” by Findley et al. (9) discusses how the human microbiome is influenced by social and physical environments in addition to biological systems, which likely act collectively to inform disease progression. The authors discuss several studies that have examined differences in the microbiomes of “ethnically diverse” cohorts but are critically limited due to the lack of social, behavioral, and environmental data on study participants. It then describes a conceptual framework for human health and disease that “acknowledges three overlapping but distinct and complex areas of health research – the microbiome, biological processes, and the social/physical environment.” A critical tenet of both papers is that social/environmental factors must be considered potential mediators with measurable impacts on disease processes instead of confounders.

As a Black early-career scientist studying the link between asthma and the respiratory microbiome, these frameworks have laid the foundation for my approach to research. Boyd and colleagues describe racism as the mechanism by which racial categorizations have biological consequences (10). Reflecting on my childhood, the link between structural racism and my elementary school district comes into glaring focus. In the late 90s (and today), my school and the surrounding district were approximately 95% nonwhite. My school was also in a county with the highest rate of asthma hospitalizations compared to surrounding counties, and recent evidence has finally uncovered the severe environmental harms caused to the community, particularly, extremely high levels of airborne toxins and pollutants and an increased cancer risk far above the national average (11).

Human microbial diversity (much like genetic diversity) is expansive and irreverent to our constructed social categories. Therefore, as microbiologists, we must be conscious of how microbiome studies that focus on minoritized populations and/or health disparities are framed. Currently, an unspoken single group (cis, hetero, white, healthy, with access to health care) is considered the standard, while the clinical or microbial features and outcomes of others are considered aberrations or even diseased. This approach furthers the notions of biological superiority/inferiority that have historically driven the scientific and medical discourse. As a scientific community, we must reconstruct our methodologies to uproot these racist practices. As an asthma researcher, this means interrogating the host, environmental, and lifestyle data we collect. A larger or more diverse study cohort is not sufficient. We must also collect data to further understand our study participants and their environmental context: physical environment, stress (economic and psychological), social factors (poverty, segregation, exposure to violence), quality health care access, and other variables to interrogate their impacts on the microbiome.

Undergoing this conceptual shift and implementing these models will be challenging. It will likely require the development of innovative statistical approaches and methods by cross-disciplinary research collaborations and community partnerships to integrate these complex, multifaceted data. It also requires a commitment by funding agencies to facilitate these studies and validate this work as crucial to our progress in science, medicine, and public health. Finally, scientific publishers must hold microbiome studies accountable in integrating the biopsychosocial model, thus affirming the broad relevance of these scholarly discussions and pushing the scientific enterprise toward equity.
